# Engineering herbicide resistance via prime editing in rice

**DOI:** 10.1111/pbi.13399

**Published:** 2020-06-11

**Authors:** Haroon Butt, Gundra Sivakrishna Rao, Khalid Sedeek, Rashid Aman, Radwa Kamel, Magdy Mahfouz

**Affiliations:** ^1^ Laboratory for Genome Engineering and Synthetic Biology King Abdullah University of Science and Technology (KAUST) Thuwal Saudi Arabia

**Keywords:** prime editing, homology‐directed repair, herbicide resistance, genome engineering

Although CRISPR‐Cas9 has revolutionized our ability to generate site‐specific double‐strand breaks, precise editing of the genome remains challenging in most eukaryotes, including plants (Shan *et al*., 2013). In plants, homology‐directed repair is inefficient, limiting our ability to make precise edits of the DNA sequence (Ali *et al*., 2020; Butt *et al*., 2017). Moreover, cytosine and adenine base editors have serious drawbacks including lower efficiency, unclean edited sequence and the possibility of off‐target mutations at other loci (Rees and Liu, 2018). Chimeric single‐guide RNAs (sgRNAs) can provide editing information, in RNA form, but this modality suffers from several limitations including lower efficiency, less versatility and the need for long homology arms (Butt *et al*., 2017).

In contrast to genome editing methods that use just a Cas nuclease to generate double‐strand breaks, prime editing employs a Cas9 nickase (nCas9) fused with reverse transcriptase (RT). The desired edits are encoded on a prime editing guide RNA, which guides the nCas9‐RT complex to the target site (Anzalone *et al*., 2019). There, the nCas9 generates a single‐strand break (Shrivastav *et al*., 2008) on the non‐complimentary strand and the RT domain transfers the desired edits from the pegRNA to the DNA (Anzalone *et al*., 2019). Researchers have developed several prime editing strategies: in PE1, wild type M‐MLV RT fused to the C terminus of Cas9 (H840A) nickase; in PE2, Cas9 (H840A) with pentamutant M‐MLV RT (D200N/ L603W/ T330P/ T306K/ W313F); in PE3, a PE2 prime editor with additional simple gRNA to simultaneously nick the non‐edited strand (Anzalone *et al*., 2019). Prime editing has several advantages over other methods, such as enabling precise sequence deletion, addition and substitution. However, although it has been tested in human cell lines, prime editing remains to be tested in plants.

To test prime editing in rice (*Oryza sativa*), we first attempted to engineer herbicide resistance by targeting rice *ACETOLACTATE SYNTHASE* (*OsALS*). ALS catalyses the initial step common to the biosynthesis of the branched‐chain amino acids and is primary target site for herbicides like Bispyribac sodium. A single amino acid change (W548L) in ALS results in a BS‐resistant phenotype (Butt *et al*., 2017). We cloned the PE2 fragment containing Cas9 (H840A) with pentamutant M‐MLV RT under the control of the *OsUBIQUITIN* promoter in rice vectors. We therefore designed a pegRNA to edit the *OsALS* sequence. The RT template with a length of 15 bp has two substitutions, a G‐to‐T substitution that converts tryptophan 548 to leucine and a silent G‐to‐A substitution that destroys the PAM site thus preventing re‐targeting by the pegRNA‐nCas9‐RT machinery (Figure 1a). These nucleotide modifications result in the loss of a BsaXI site and generation of an MfeI site. The primer binding site (PBS) was designed with a length of 13 bp. The pegRNA was expressed in rice vectors under the *OsU3* promoter.

**Figure 1:**
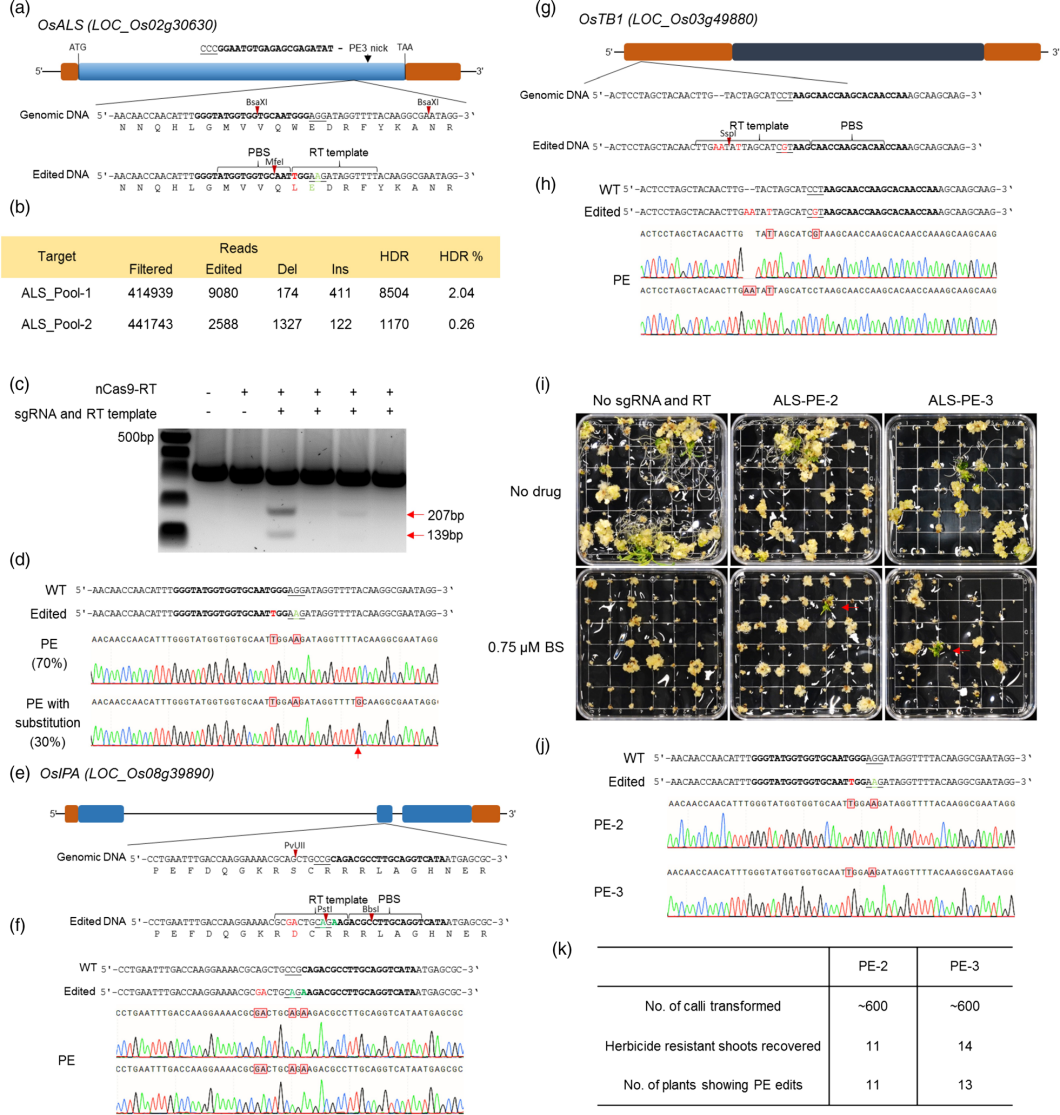
Prime editing of *OsALS* for herbicide resistance. (a) Schematic representation of rice *ALS* locus. A single nucleotide substitution TGG to TTG (W548L) produced a herbicide resistance in rice. The repair template (RT) designed with two substitutions, G to T for herbicide resistance and a silent mutation G to A to destroy PAM site. These substitutions generate MfeI site and abolish the BsaXI site. The exon is indicated as blue box. (b) The rice callus was transformed with ALS‐PE2 (nCas9‐RT_pegRNA) via agrobacterium. After two rounds of selections of T‐DNA on hygromycin, the proliferating rice calli were pooled and used for amplicon deep sequencing. (c) The amplicons from different rice calli were enriched for editing by BsaXI and after purification, PCR was done and amplicons were digested with MfeI. The digested production indicates the editing in the cells and further confirmed via Sanger sequencing (d). Some of the edited reads, indicated by arrow, were also showing A to G substitutions. This G probably corresponds to the first base of the scaffold RNA adjacent to RT template in pegRNA. (e) Schematic of rice locus *IPA* (*Ideal Plant Architecture*). We have designed a pegRNA for two consecutive substitutions AG to GA to convert S163 to D. Two silent substitutions have been done CGC to AGA which convert R165 to R and destroy the PAM site. By these mutations, PvUII site was lost and two sites Pst1 and BbsI were generated. (f) The PvUII enriched DNA samples are confirmed by Sanger sequencing. (g) Schematic of rice locus *TB1* (*TEOSINTE BRANCHED1*). We have designed a pegRNA to target the GTAC motif in promoter of OsTB1. In the repair, template C was converted to G to destroy PAM site. Two consecutive insertions AA and one substitution were done to destroy binding motif. These mutations also created SspI restriction site and destroy RsaI site. (h) The RsaI enriched DNA samples are confirmed by Sanger sequencing. (i) The ALS‐PE‐2 and ALS‐PE‐3 plasmids were transformed in rice via agrobacterium. After selection, the regeneration was done with 0.75 µm BS. Arrows indicate the regeneration of herbicide‐resistant shoots. (j) The selected PCR fragments were analysed by Sanger sequencing. (k) The number of plants recovered from PE‐2 and PE‐3 is almost equal. Some of the herbicide resistance plants are homozygous.

We transformed rice via *Agrobacterium* and after two weeks of selection, we collected four independently growing calli from different selection plates. We performed the DNA extraction from these calli and amplified the target DNA by PCR. We pooled the amplicons in equimolar concentrations and performed deep sequencing. Our data showed that the prime editing successfully edited *OsALS* at the target site with an efficiency of 0.26 to 2% (Figure 1b). The different editing efficiencies among two pools are possibly due to varied number of non‐edited WT cells between these calli. The editing efficiencies are further validated when we enriched the edited DNA from the four calli by cutting with BsaXI (which cuts the unedited sequence) and conducted PCR/restriction enzyme analysis (PCR/RE) using MfeI (Figure 1c). The digestion of amplicons by MfeI indicated the frequency of editing in the samples. We used Sanger sequencing to confirm these edits (Figure 1d). Most of the reads were fully edited and repaired according to the RT template. Interestingly, some of the reads showed an A‐to‐G substitution, which converts tyrosine 553 to cysteine. This substitution is not the part of the RT template and probably came from the scaffold RNA, as the first nucleotide of the scaffold RNA adjacent to the RT template (a ‘G’) can be used for DNA repair (Figure 1d).

We also targeted rice *IDEAL PLANT ARCHITECTURE 1* (*OsIPA*) using prime editing (Figure 1e). The OsIPA transcription factor reduces the number of unproductive tillers and improves rice yield. We designed a pegRNA for two consecutive substitutions (AG to GA) to convert S163 to D in IPA with length of RT 20 bp and PBS 13 bp. Two silent substitutions (CGC to AGA) destroy the PAM site. These mutations destroy a PvuII site and generate Pst1 and BbsI sites. We transformed rice via *Agrobacterium* and regenerated shoots. We analysed the plantlets after enriching for edited DNA with PvuII digestion by Sanger sequencing. We found that prime editing successfully edited *OsIPA* at the target site, (Figure 1f).

Similarly, we targeted rice *TEOSINTE BRANCHED 1* (*OsTB1*), a member of the *TEOSINTE BRANCHED1, CYCLOIDEA AND PCF TRANSCRIPTION FACTOR* gene family (Figure 1g). OsTB1 negatively regulates lateral branching by repressing axillary bud outgrowth. We designed a pegRNA to target the *OsTB1* promoter with length of RT 20 bp and PBS 13 bp. A C‐to‐G substitution destroyed the PAM to prevent re‐targeting and two consecutive insertions (AA) and one substitution (C to T) destroyed and RsaI site and created an SspI site. We analysed the shoots by enriching the DNA with RsaI digestion and by Sanger sequencing and observed partial repair and different types of reads (Figure 1h). The possible reason for chimeric cells is that prime editing machinery could be still functional in the non‐edited cells and continuously modified the targeted region.

To test whether we could improve the editing efficiency, we tried the PE3 strategy, where a second sgRNA is used to nick the complimentary strand. We designed the sgRNA to target *OsALS* at a distance of +55 from the pegRNA and expressed this sgRNA from the *OsU3* promoter using the polycistronic tRNA‐gRNA system (Butt *et al*., 2017; Xie *et al*., 2015) (Figure 1a). We transformed the rice callus with ALS‐PE2 (containing just the pegRNA and RT‐nCas) and ALS‐PE3 (containing the sgRNA, pegRNA, and RT‐nCas) plasmids. After selection, we regenerated shoots on media supplemented with 0.75 µm BS (Figure 1i). For both ALS‐PE2 and ALS‐PE3, we recovered shoots resistant to BS. Sanger sequencing showed that these plantlets were successfully edited (Figure 1j). We recovered almost equal numbers of shoots from PE2 and PE3 (Figure 1k), suggesting that (unlike mammalian systems) PE3 did not increase editing efficiency in plants.

In the present study, we successfully used prime editing technology on three loci in plants. While this work was prepared for publication, similar findings were reported in pants (Li *et al*., 2020; Lin *et al*., 2020; Tang *et al*., 2020). We engineered herbicide resistance trait in rice via nucleotide substitutions; however, the system requires further improvements and assessments on its ability to enable diverse editing modalities for different trait engineering applications in plants.

## Funding

This work is funded by KAUST‐baseline funding to Magdy Mahfouz.

## Competing interests

The authors declare that they have no competing interests.

## Author contributions

MM conceived the project; HB, GSR, KS, RA and RK conducted the experiments; HB, GSR, KS, RA and MM analysed the data; and HB, GSR and MM wrote the manuscript.

## References

[pbi13399-bib-0001] Ali, Z. , Shami, A. , Sedeek, K. , Kamel, R. , Alhabsi, A. , Tehseen, M. , Hassan, N. *et al*. (2020) Fusion of the Cas9 endonuclease and the VirD2 relaxase facilitates homology‐directed repair for precise genome engineering in rice. Commun Biol. 3, 44. 31974493 10.1038/s42003-020-0768-9 PMC6978410

[pbi13399-bib-0002] Anzalone, A.V. , Randolph, P.B. , Davis, J.R. , Sousa, A.A. , Koblan, L.W. , Levy, J.M. , Chen, P.J. *et al*. (2019) Search‐and‐replace genome editing without double‐strand breaks or donor DNA. Nature, 576, 149–157. 31634902 10.1038/s41586-019-1711-4 PMC6907074

[pbi13399-bib-0003] Butt, H. , Eid, A. , Ali, Z. , Atia, M.A.M. , Mokhtar, M.M. , Hassan, N. , Lee, C.M. *et al*. (2017) Efficient CRISPR/Cas9‐mediated genome editing using a chimeric single‐guide RNA molecule. Front Plant Sci. 8, 1441–1441. 28883826 10.3389/fpls.2017.01441 PMC5573723

[pbi13399-bib-0004] Li, H. , Li, J. , Chen, J. , Yan, L. and Xia, L. (2020) Precise modifications of both exogenous and endogenous genes in rice by prime editing. Mol Plant, 13, 671–674. 32222486 10.1016/j.molp.2020.03.011

[pbi13399-bib-0005] Lin, Q. , Zong, Y. , Xue, C. , Wang, S. , Jin, S. , Zhu, Z. , Wang, Y. *et al*. (2020) Prime genome editing in rice and wheat. Nat Biotechnol. 38, 582–585. 32393904 10.1038/s41587-020-0455-x

[pbi13399-bib-0006] Rees, H.A. and Liu, D.R. (2018) Base editing: precision chemistry on the genome and transcriptome of living cells. Nat. Rev. Genet. 19, 770–788. 30323312 10.1038/s41576-018-0059-1 PMC6535181

[pbi13399-bib-0007] Shan, Q. , Wang, Y. , Li, J. , Zhang, Y. , Chen, K. , Liang, Z. , Zhang, K. *et al*. (2013) Targeted genome modification of crop plants using a CRISPR‐Cas system. Nat. Biotechnol. 31, 686–688. 23929338 10.1038/nbt.2650

[pbi13399-bib-0008] Shrivastav, M. , De Haro, L.P. and Nickoloff, J.A. (2008) Regulation of DNA double‐strand break repair pathway choice. Cell Res. 18, 134–147. 18157161 10.1038/cr.2007.111

[pbi13399-bib-0009] Tang, X. , Sretenovic, S. , Ren, Q. , Jia, X. , Li, M. , Fan, T. , Yin, D. *et al*. (2020) Plant prime editors enable precise gene editing in rice cells. Mol Plant, 13, 667–670. 32222487 10.1016/j.molp.2020.03.010

[pbi13399-bib-0010] Xie, K. , Minkenberg, B. and Yang, Y. (2015) Boosting CRISPR/Cas9 multiplex editing capability with the endogenous tRNA‐processing system. Proc. Natl Acad. Sci. USA, 112, 3570–3575. 25733849 10.1073/pnas.1420294112 PMC4371917

